# Miscellaneous Intelligence

**Published:** 1800-04

**Authors:** Benj. Hutchinson

**Affiliations:** Southwell, Nott.


### *Miscellaneous Intelligence.*

To the Editors of the Medical and Physical Journal.

Gentlemen,

I SHALL esteem it a favor, if you will insert the following article in your valuable Work; and am,

Your obedient humble servant,

*Southwell, Nott.*
*Mar.* 8, 1800.

BENJ. HUTCHINSON.

Dr. Soemmering, of Frankfort on the Mayne, having read the Life of Mr. Charles Darwin in my *Biographia Medica*, (in which a passage is quoted from an ingenious Thesis on Hectic Fever, by Dr. Cappe, of York) thinks himself ill used in being accused of publishing a falsity, by saying in a Treatise of his on Diabetes, that Mr. Charles Darwin, had not, in fact, made the experiments on Pus and Mucus, for which the first prize medal was allotted to him at Edinburgh, but that he wrote those experiments from imagination;—Dr. Soemmering, in a letter which I lately received from him, asserts that he was so informed by Mr. Fyfe, and hopes the passage alluded to in my Biographia Medica, may be contradicted in some respectable publication, or otherwise omitted in a future edition of that work.

I have therefore troubled you with this account, but beg to add, that Dr. Cappe of York, and Dr. Ryan of Dublin, repeated Mr. Charles Darwin's experiments, and found similar results with those described by him; and also, that Dr. Soemmering must still continue the propagator, though not the inventor, of the assertion in his Treatise on Diabetes. This circumstance cannot reflect any credit on the very ingenious German physiologist, as the contrary facts ought to be established by repeated experiments, not by hearsay evidence.

---

Messrs. Tennant and Pearson having announced that they had succeeded in obtaining carbon by decomposing the carbonat of lime by means of phosphorus, the Society, in consequence of this assertion, requested Citizens Vauquelin and Brongniart, in conjunction with their Secretary, to repeat this experiment. Cit. Brongniart shortly after read a Memoir on the theory of the different affinities of oxygen with carbon, phosphorus, and with the phosphoric and carbonic acids combined combined with alkalies; in which he proved, that the fact announced by the English physicians was founded on experience. The members of the Society, charged with this commission, consequently presented a considerable quantity of carbon obtained from the decomposition of the carbonats of lime and soda, by means of phosphorus.—*Rapport General des Travaux de la Société Philomatique de Paris*, An. vi. p. 51.

---

Cit. Brongniart read a Memoir to the Society, on the characters to be adopted in describing mineral substances. He applied simple characters to each of the great divisions of this kingdom, and marked the species and varieties by particular additions to these signs; so that they present a systematic division very analogous to that adopted for mineralogical science, and the fertility of which, in all their possible variations, surpasses that of all natural substances known in the mineral kingdom. —*Ibid.* p. 82.

---

The same member read some passages from his Journey to the Alps, in which he had inserted some observations on the natural history and œconomy of those countries, and on the manners of their inhabitants. He rested his description of the specimens which contributed to confirm his opinion on the origin of several rocks which compose this chain of mountains, particularly on that of a porphyroidal pudding *(pouding porphiroide)*, which he considers as primitive, that is, of a formation coeval to that of the crystallization of the mountains called primitive. These pudding stones are contained in perpendicular banks, alternating with those of a micacious schistus, without being intermixed with other rocks. He observed, that in their composition the angles of the schistus are scarcely obtuse, while those of the quartz, which is comparatively very hard, almost uniformly appear as if they were rolled.—*Ibid.* p. 83.

---

Cit. Halle made a report to the Society, on a case of simple idiopathic atrophy. The subject of this observation was a young female, who died at the age of 25, in consequence of a considerable wasting, without any apparent cause. She had been cachectic from the fifth or sixth year of her age; at seven she had a slight menstruation, but which did not continue long; at fourteen the catemenia commenced, and from her seventeenth to her twenty-first year they gradually diminished, at which period they entirely stopped, and she became progressively leaner till the time of her decease; though she was enabled to follow her usual employments, without observing any difference in her evacuations. Her complaint terminated without any other symptoms fymptoms than laffitude, weaknefs, and an inclination to fleep. On opening the body, the fkin appeared to adhere to the bones. The abdomen was depreffed, and almoft touched the vertebræ; and no appearance of fat could be obferved either in the epiploon or the mefentery. On raifing the fkin of the groin, he perceived feveral white and dry threads, refembling nerves, with tumefactions not unlike the nervous ganglions. It was evident that thefe were the lymphatic veffels which had changed to that ftate. The cavity of thefe veffels appeared completely obliterated, as the ufual traces of them were no where perceptible, nor could thofe of the lacteals be difcovered. No caufe could be affigned for this fingular difeafe, unlefs it were attributed to long continued and oppreffive mental affections, which were carefully concealed.—*Ibid.* p. 128.

---

Cit. CUVIER read a Memoir on the circulation of the blood in fuch animals as have this fluid of a white colour. After having taken a view of the different combinations eftablifhed by Nature, with refpect to the organs of circulation in the various claffes of animals, he obferves, that thofe which have white blood are provided but with one order of veffels, conveying only a fimple lymph. He founds his argument particularly on the immediate communication of thofe veffels with all the cavities of the body, and obferves, that as the inteftinal canal paffes through the heart of feveral of them, the chyle which tranfudes immediately into that organ, is fufficient for the fupply of the body.—*Ibid.* p. 137.

---

The fame member read a Memoir on the manner in which nutrition apparently takes place in infects. From the opinions of feveral authors, as well as from his own obfervations, he has afcertained that the dorfal veffel of infects is not a heart, and that it poffeffes no branch which can ferve for the purpofe of circulation. The author afferts, that infects are provided with no other veffels than the trachea, that the nutritive juice paffes only through the inteftinal canal, and that all the parts derive their aliment from fimple fuction. He alfo obferves, that the fecretory organs of infects do not form folid glands, fuch as are found in all animals that are provided with a heart, but that they confift of ifolated and fpongy tubes; and, finally, that the whole organization of this clafs of animals is difpofed, as if they had neither heart nor blood veffels.—*Ibid.* p. 138.

---

Prof. HERMBSTAEDT, of Berlin, has lately made the important difcovery of feparating the mineral alkali, or foda, from common falt, by a very cheap and expeditious procefs. The king

King of Pruſſia, not inſenſible of the great advantages to be derived from this new method of obtaining ſo valuable and uſeful an article of commerce, has conſequently eſtabliſhed a chemical manufactory at Schoenbeck, where that commodity is produced in the great way, at the expence of the Crown.

---

Dr. Bouvion, a phyſician of ſome eminence in Straſburg, has written to Paris to obtain all the medical aſſiſtance that ſcience can afford, with a view to ſtop the further progreſs of an epidemical diſorder which has broken out in that city very lately, and which he confeſſes to have been treated hitherto, by the faculty of the place, without ſucceſs. The ravages of it are deſcribed to be great, and afflicting to humanity. It manifeſts itſelf by an angina of a malignant nature, complicated with a ſcarlet fever; it particularly attacks young perſons between the age of eighteen and twenty-four. The tables of the meteorologiſts of Straſburg have been conſulted by the medical gentleman, without deriving any ſatiſfaction as to the cauſe of this deplorable occurence. The period when the diſorder is fatal, is not ſo regular as in many endemical and epidemical diſeaſes.

---

The Elector of Saxony has iſſued orders for erecting a Chemical Laboratory, and an Inſtitution for the inſtruction of ſtudents in midwifery, in the Univerſities of Leipzig and Wittenberg.

---

The Botanical Society of Nuremberg has been revived by the exertions of Count Sternberg, after having ceaſed for ſeveral years to hold its meeting.

---

Mr. John Bell's Treatiſe on Wounds has been tranſlated into the German, with remarks and additions, by Dr. J. C. F. Leune, Leipzig, Bohemia.

---

